# Long-term outcomes of stereotactic body radiation therapy (SBRT) with fiducial tracking for inoperable stage I non-small cell lung cancer (NSCLC)

**DOI:** 10.1007/s13566-016-0273-4

**Published:** 2016-08-20

**Authors:** Jonathan W. Lischalk, Stephanie M. Woo, Shaan Kataria, Nima Aghdam, Ima Paydar, Michael C. Repka, Eric D. Anderson, Brian T. Collins

**Affiliations:** 1 Department of Radiation Medicine, Georgetown University Hospital, Lower Level Bles, 3800 Reservoir Road, N.W, Washington, DC 20007 USA; 2 Division of Pulmonary, Critical Care, and Sleep Medicine, Georgetown University Hospital, Pasquerilla Healthcare Center, 5th floor, 3800 Reservoir Road, N.W., Washington, DC 20007 USA

**Keywords:** Lung neoplasms, Non-small cell lung cancer, Stereotactic body radiation therapy, Pulmonary function test, Fiducial markers

## Abstract

**Background:**

Stereotactic body radiation therapy (SBRT) for stage I non-small cell lung cancer (NSCLC) is considered standard of care in the medically inoperable patient population. Multiple methods of SBRT delivery exist including fiducial-based tumor tracking, which allows for smaller treatment margins and avoidance of patient immobilization devices. We explore the long-term clinical outcomes of this novel fiducial-based SBRT method.

**Methods:**

In this single institutional retrospective review, we detail the outcomes of medically inoperable pathologically confirmed stage I NSCLC. Patients were treated with the Cyberknife SBRT system using a planning target volume (PTV) defined as a 5-mm expansion from gross tumor volume (GTV) without creation of an internal target volume (ITV). Dose was delivered in three or five equal fractions of 10 to 20 Gy. Pretreatment and posttreatment pulmonary function test (PFT) changes and evidence of late radiological rib fractures were analyzed for the majority of patients. Actuarial local control, locoregional control, distant control, and overall survival were calculated using the Kaplan-Meier method.

**Results:**

Sixty-one patients with a median age of 75 years were available for analysis. The majority (80 %) of patients were deemed to be medically inoperable due to underlying pulmonary dysfunction. Eleven patients (18 %) developed symptomatic pneumothoraces secondary to fiducial placement under CT guidance, which precipitously dropped to 0 % following transition to bronchoscopic fiducial placement. The 2-year rib fracture risk was 21.4 % with a median time to rib fracture of 2.9 years. PFTs averaged over all patients and parameters demonstrated small absolute declines, 5.7 % averaged PFT decline, at approximately 1 year of follow-up, but only the diffusing capacity of lung for carbon monoxide (DL_CO_) demonstrated a statistically significant decline (10.29 vs. 9.01 mL/min/mmHg, *p* = 0.01). Five-year local control, locoregional control, and overall survival were 87.6, 71.8, and 39.3 %, respectively.

**Conclusions:**

Despite reduced treatment margins and lack of patient immobilization, SBRT with fiducial-based tumor tracking achieves clinically comparable long-term outcomes to other linac-based SBRT approaches.

## Introduction

The 2016 incidence of newly diagnosed lung cancer is estimated to be 224,390 with approximately 158,080 of these patients succumbing to their disease [1]. Lung cancer continues to be the leading cause of cancer-related death for both men and women in the United States and is the leading cause of cancer death worldwide, accounting for 18 % of all cancer-related deaths [2–4]. Non-small cell lung cancer (NSCLC) comprises the vast majority of cases (∼85 %), with adenocarcinoma being the most common histologic subtype [5]. Unfortunately, the majority of patients are diagnosed with metastatic disease at presentation [2]. These abysmal statistics prompted the National Lung Screening Trial Research Team to explore the utility of low-dose computed tomographic (CT) screening in a high-risk population [6]. The results demonstrated low-dose CT screening yielded a 20 % relative reduction in mortality from lung cancer and a 6.7 % relative reduction in all-cause mortality [6]. These practice changing results prompted the US Preventive Services Task Force in 2013 to recommend annual screening for lung cancer with low-dose CT scans in patients aged 55 to 80 with a 30 pack-year smoking history who are current smokers or quit within the past 15 years [7]. As implementation of these new screening guidelines becomes ubiquitous, an increase in the incidence of early-stage NSCLC is plausible.

The current National Comprehensive Cancer Network (NCCN) standard of care for medically operable stage I NSCLC continues to be anatomical surgical resection, preferably a lobectomy. Increasing proportions, estimated at 25 % of patients, however, are considered inoperable either due to advanced age or significant medical comorbidities precluding thoracic surgery [8]. Historically, conventionally fractionated radiotherapy yielded low rates of overall survival of approximately 15 % and widely variable local control ranging from 30 to 70 % [8, 9]. However, the development of stereotactic body radiation therapy (SBRT) as a treatment alternative to conventional fractionation led to the landmark phase II RTOG 0236 trial, which demonstrated a marked improvement in 3-year local control and overall survival of 98 and 56 %, respectively [10].

A number of modalities of SBRT delivery have since been established, including one unique modality, which utilizes fiducial-based tumor tracking. This technique uses gold markers placed by an interventional pulmonologist within or directly adjacent to the tumor to monitor lesion movement with respiration using periodically obtained x-rays during treatment. The non-isocentric robotic stereotactic system allows for highly conformal dose distributions with rapid dose fall off, permitting treatment margin reduction and thus minimizing delivery of excess dose to adjacent normal tissues [11, 12]. The aggregate result is a minimization of excess dose to adjacent lung parenchyma, heart, and esophagus as well as avoidance of burdensome patient immobilization devices during treatment. Since 2005, our institution has treated patients with this image-guided fiducial-based SBRT technique utilizing continuous tracking of tumor motion [11, 12]. In this retrospective analysis, we investigate clinical outcomes of patients with stage I NSCLC from a single institution treated with robotically delivered fiducial-based SBRT.

## Materials and methods

### Patient eligibility

The local Health Research Institutional Review Board (IRB) approved this retrospective analysis of an established departmental treatment approach. Inclusion criteria for this investigation included (1) pathologic confirmation of NSCLC malignancy, (2) American Joint Committee on Cancer (AJCC) seventh edition stage I (cT1a to cT2a) disease, (3) pretreatment staging PET/CT scan, (4) acceptable fiducial placement for safe SBRT delivery as determined by the attending radiation oncologist, and (5) medical inoperability determined by a single pulmonologist (EDA). In general, patients were deemed medically inoperable if they demonstrated preoperative forced expiratory volume (FEV1) <40 % of predicted value, diffusing capacity of lung for carbon monoxide (DL_CO_) <40 % of predicted value, very poor exercise tolerance, and/or significant underlying cardiac disease. However, operability was determined on a case-by-case basis. Exclusion criteria included (1) prior history of malignancy within the last 5 years, (2) prior thoracic radiation, (3) prior systemic therapy, and (4) identification of any other suspicious pulmonary nodules. All patients were evaluated and underwent placement of fiducials via CT guidance or electromagnetic navigational bronchoscopy within or directly adjacent to the tumor.

### Treatment planning and delivery

A fine-cut treatment planning CT scan was obtained in the supine treatment position for each patient using a Light-Speed RT16 (GE Healthcare, Little Chalfont, United Kingdom). Full-inhalation CT scans were obtained with intravenous contrast, oral contrast, and/or esophageal paste when clinically indicated. PET/CT scans, requisite for study inclusion, were obtained for staging and treatment planning purposes. The gross tumor volume (GTV) was contoured using lung window settings and with input from the attending pulmonologist. A 5-mm expansion was added to the GTV to generate a planning target volume (PTV). Of note, the CyberKnife Synchrony System (Cyberknife, Sunnyvale, CA, USA) of intrafractional tumor motion tracking precluded the creation of an internal target volume (ITV). A treatment plan was generated using the MultiPlan 5.2.1 non-isocentric inverse-planning algorithm. Radiation was delivered in three or five equal fractions of 10 to 20 Gy prescribed to an isodose line that covered at least 95 % of the PTV. All dose fractionation schedules were assured to deliver a biological effective dose (BED) of at least 100 Gy using an NSCLC tumor α/β of 10 Gy. Patients were treated in the supine position with arms at their sides without immobilization device utilization. The Synchrony Respiratory Motion Tracking System was used to accommodate for patient-specific respiratory motion during treatment [11, 13]. Treatment was typically delivered over consecutive days, usually over one week and not exceeding two weeks.

### Follow-up

Patients were followed with physical examination and CT with or without PET imaging at 3-to 6-month intervals per routine institutional practice. Pulmonary function tests (PFTs) were obtained for the majority of patients before and after radiotherapy. Post-treatment PFTs were obtained at least 90 days following radiotherapy and if possible, at approximately one year of follow-up. PFT paired comparisons were made only on patients with available pre-treatment and post-treatment PFTs. Follow-up CT scans were used to detect radiological rib fractures and were reviewed by two radiation oncologists (BTC and JWL). Local tumor recurrence was defined as documented tumor progression within the treated field (i.e. in-field failures) as evaluated on follow-up imaging. Locoregional failure was defined as any failure within the treated field, involved lobe, or ipsilateral nodal region (N1 to N2). Each recurrence was reviewed and confirmed by the same two radiation oncologists (BTC and JWL). Local control, locoregional control, and distant control were measured from the date of treatment completion to the date of last radiological follow-up, radiological progression, or death. Patients who were alive without failure were censored at the date of last radiological follow-up. Overall survival was measured from the date of treatment completion to the date of patient death.

### Statistical analysis

Statistical analysis was performed with the IBM SPSS Statistics version 22 (IBM Corporation and other(s) 1989, 2013, Armonk, NY, USA). Comparison of paired cohort-averaged PFT changes before and after radiotherapy was performed using the Wilcoxon signed rank test. Actuarial local control, locoregional control, distant control, and overall survival were calculated using the Kaplan-Meier method. Univariate analysis of factors affecting local control, locoregional control, distant control, and overall survival were determined using generalized Wilcoxon analysis.

## Results

### Patient and tumor characteristics

Sixty-one patients with a median age of 75 years (range, 59 to 94 years) were treated from August 2005 to September 2015. The vast majority of patients attested to a history of tobacco use (93 %) with a median pack-year smoking history of 41.0. Of those with a smoking history, 77 % were classified as former smokers and 23 % as current smokers. Surgical inoperability was primarily due to prohibitive pulmonary dysfunction in 80 % of cases with the remainder due to advanced age and/or other medical comorbidities. In fact, 31 % of all patients had baseline oxygen dependency. The majority of tumors were located within the left upper lobe (30 %) and right upper lobe (26 %). The mean maximum tumor diameter on pretreatment CT scan was measured to be 2.5 cm (range, 0.9 to 5.0 cm). The majority of tumors treated were AJCC seventh edition prognostic stage IA (75 %). Median tumor SUV_max_ on pretreatment PET scan was 5.7. Specific patient and tumor characteristics are shown in Tables 1 and 2.

**Table 1:** Patient characteristics

Characteristic	No. of patients	Percent
Age		
≤75	31	51
>75	30	49
Gender		
Male	20	33
Female	41	67
ECOG performance status		
0	4	7
1	28	46
2	22	36
3	7	11
Smoking status		
Never	4	7
Former	44	72
Current	13	21
Tobacco history (pack-years)		
0	4	7
<40	20	33
40–50	21	34
>50	16	26
Baseline oxygen dependence		
Yes	19	31
No	42	69
Reason for inoperability		
Age	11	18
Pulmonary dysfunction	49	80
Other medical comorbidities	1	2

**Table 2:** Tumor characteristics

Characteristic	No. of patients	Percent
Histology		
Adenocarcinoma	30	49
Squamous cell carcinoma	16	26
Unspecified NSCLC	15	25
Tumor max diameter (cm)		
<2	20	33
2–3	26	43
3–5	15	24
AJCC prognostic stage		
IA	46	75
IB	15	25
PET max SUV		
<4	19	31
4–10	28	46
>10	13	23
Tumor lobe location		
RUL	16	26
RML	1	2
RLL	11	18
LUL	18	30
LLL	15	24

### Treatment characteristics

Thirty-two patients (52 %) underwent placement of fiducials via electromagnetic navigational bronchoscopy with the remaining 29 (48 %) under CT guidance. Three to four fiducials were placed in the majority (81 %) of cases. The number of fiducials placed was at the discretion of the interventional pulmonologist with the understanding that a minimum of three fiducials was required for rotational tracking. Patients were treated using the CyberKnife robotic radiosurgical system to a median total dose of 50 Gy (range, 45 to 60 Gy) with a median BED of 112.5 Gy (range, 100.0 to 180.0 Gy). The median number of treatment fractions and dose per fraction was 5 (range, 3 to 5) and 11 Gy/fraction (range, 10 to 20 Gy/fraction), respectively. Median treatment duration from start to completion of SBRT was 7 days (range, 3 to 15 days). Mean and median GTV was 17.8 and 13.4 cc (range, 1.3 to 62.2 cc), respectively. Treatment was delivered to a median prescription isodose line of 79 % with a median PTV target coverage of 99 %. Specific treatment characteristics are shown in Table 3.

**Table 3:** Treatment characteristics

Characteristic	No. of patients	Percent
Fiducial placement technique		
CT guided	29	48
Bronchoscopic	32	52
Number of fiducials placed		
2	7	11
3	26	43
4	23	38
Other	5	8
PTV volume (cc)		
<30	25	41
30–60	24	39
>60	12	20
Total dose (Gy)/number of fractions		
60/3	5	8
45/3	9	15
54/3	8	13
50/5	30	49
60/5	3	5
Other	6	10

### Treatment toxicity

Pneumothoraces requiring chest tube placement occurred in 11 patients (18 %), all of which were secondary to fiducial placement under CT guidance. This high rate of pneumothoraces prompted an institutional shift to utilize bronchoscopy for fiducial placement in the latter half of the study duration. There were subsequently no chest tube placements observed following the adoption of bronchoscopic fiducial placement in this cohort. Relative to CT-guided fiducial placement, electromagnetic-navigated bronchoscopic placement of fiducials resulted in minimal acute toxicity. Although cough and hemoptysis are not uncommon following bronchoscopy, the available medical records incompletely addressed these low-grade toxicities. A total of 19 rib fractures were identified on radiological follow-up. Each rib fracture was confirmed to be adjacent to or within the SBRT treatment field. The median time to rib fracture was 2.9 years with an estimated 2-year rib fracture risk of 21.4 %. In those patients with follow-up greater than 3.2 years, the rib fracture risk appeared to plateau, as no new radiological rib fractures were identified.

PFTs were performed and available for our analysis on 56 patients (92 %) prior to and 47 patients (77 %) following radiotherapy treatment. Pretreatment PFTs were obtained at a median of 56 days prior to SBRT; posttreatment PFTs were obtained at a median of 368 days following radiotherapy. Cohort-averaged FEV1, forced vital capacity (FVC), and DL_CO_ all demonstrated modest absolute decreases following radiotherapy treatment. However, DL_CO_ demonstrated the only statistically significant decrease following SBRT treatment (10.3 vs. 9.0 mL/min/mmHg, *p* = 0.01). Cohort-averaged pulmonary function tests performed prior to and following radiotherapy are detailed in Table 4.

**Table 4:** Pulmonary function test changes

Characteristic	Mean pretreatment	Mean posttreatment	Median change	*p* value
FEV1				
Liters	1.26	1.18	−0.05	0.46
% predicted	56.8	55.0	−5	0.71
FVC				
Liters	2.29	2.07	−0.06	0.45
% predicted	77.8	76.7	−3	0.47
DLCO				
mL/min/mmHg	10.29	9.01	−1.25	0.01
% predicted	53.5	52.1	−10	0.10
TLC				
Liters	5.63	5.67	−0.25	0.36
% predicted	111	134	−5	0.75

### Local control and overall survival

**Fig. 1:**
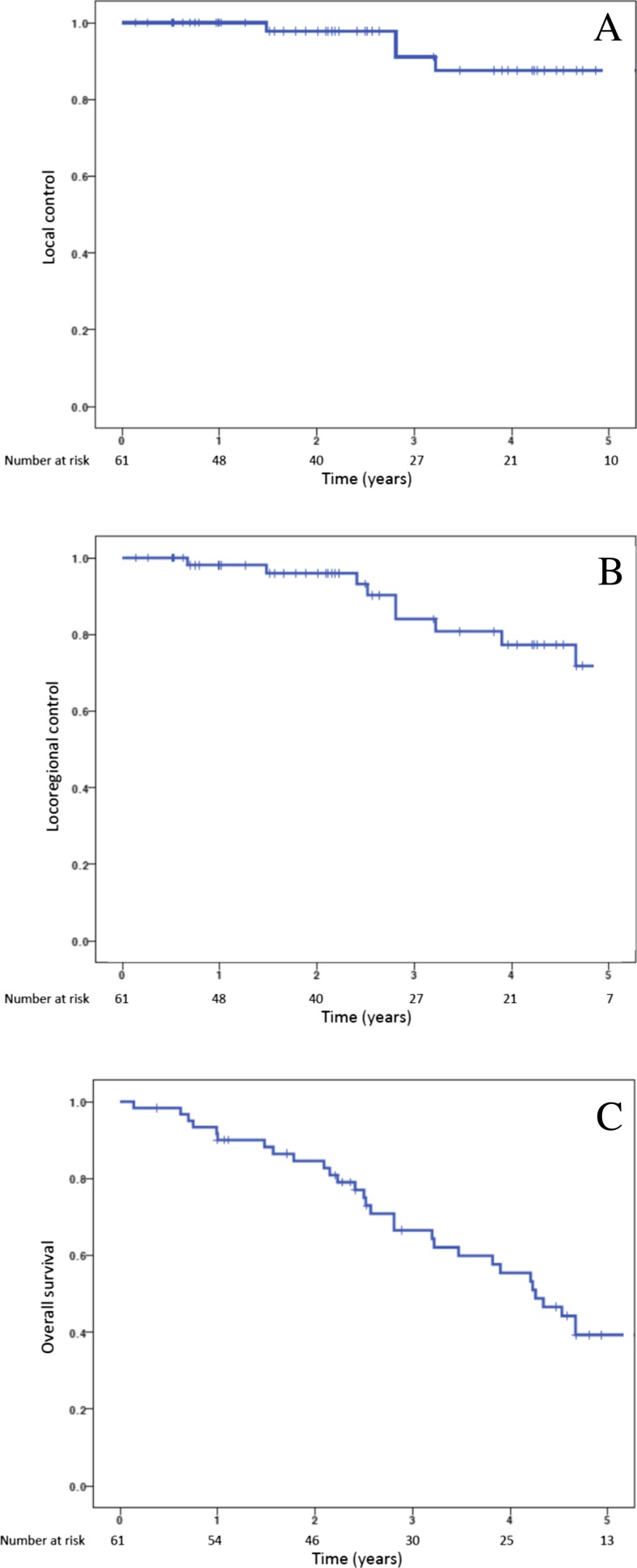
**a** Kaplan-Meier local control. **b** Kaplan-Meier locoregional control. **c** Kaplan-Meier overall survival

The 3- and 5-year Kaplan-Meier local control was 91.1 and 87.6 %, respectively (Fig. 1a). Four local recurrences were observed with a mean time to local recurrence of 2.6 years. Of note, in those patients who recurred locally, progressive local disease was eventually determined to be the cause of death in all four patients. Kaplan-Meier 3- and 5-year locoregional control was 84.1 and 71.8 %, respectively (Fig. 1b). Only one involved lobe non-local failure was observed. A total of four ipsilateral nodal failures were identified with a mean time to nodal failure of 2.4 years following treatment. At the time of analysis, three patients with regional failures had died; two patients went on to die of progressive metastatic disease, and the remaining death was due to progressive COPD.

The 3- and 5-year Kaplan-Meier distant control rate was 95.8 and 79.5 %, respectively. Of note, a total of six patients developed metastatic disease as the site of first treatment failure. A total of five patients eventually developed metachronous primaries, which included three lung, one pancreatic, and one gynecologic. The 3- and 5-year Kaplan-Meier overall survival was 66.6 and 39.3 %, respectively (Fig. 1c). The median Kaplan-Meier overall survival was estimated to be 4.3 years. Univariate analysis demonstrated squamous cell histology to be the only statistically significant predictor of local failure. Locoregional failure was significantly associated with ECOG performance status ≥2 and tumor size >2.5 cm. Only ECOG performance status ≥2 and tumor size >2.5 cm were associated with a decrement in overall survival. The patient population was undersized to detect statistically significant differences on multivariate analysis. Univariate analysis is detailed in Table 5.

**Table 5:** Univariate analysis

Parameter	LC *p*	LRC *p*	DC *p*	OS *p*
Sex				
Male vs. female	0.70	0.44	0.19	0.70
Age (years)				
>75 vs. ≤75	0.23	0.19	0.19	0.29
ECOG				
≥2 vs. <2	0.08	0.004^a^	0.13	0.03^a^
Tobacco (pack-years)				
>40 vs. ≤40	0.41	0.06	0.96	0.31
Tumor size (cm)				
>2.5 vs. ≤2.5 cm	0.23	0.04^a^	0.32	0.01^a^
PET SUV_max_				
>6 vs. ≤6	0.88	0.89	0.26	0.86
Histology				
Squamous vs. others	0.003^a^	0.30	0.76	0.54
Tumor location				
Lower vs. upper lobe	0.45	0.30	0.26	0.50
PTV (cc)				
>50 vs. ≤50	0.72	0.95	0.46	0.10

*LC* local control, *LRC* locoregional control, *DC* distant control, *OS* overall survival

^a^Statistically significant results

## Discussion

**Fig. 2.**
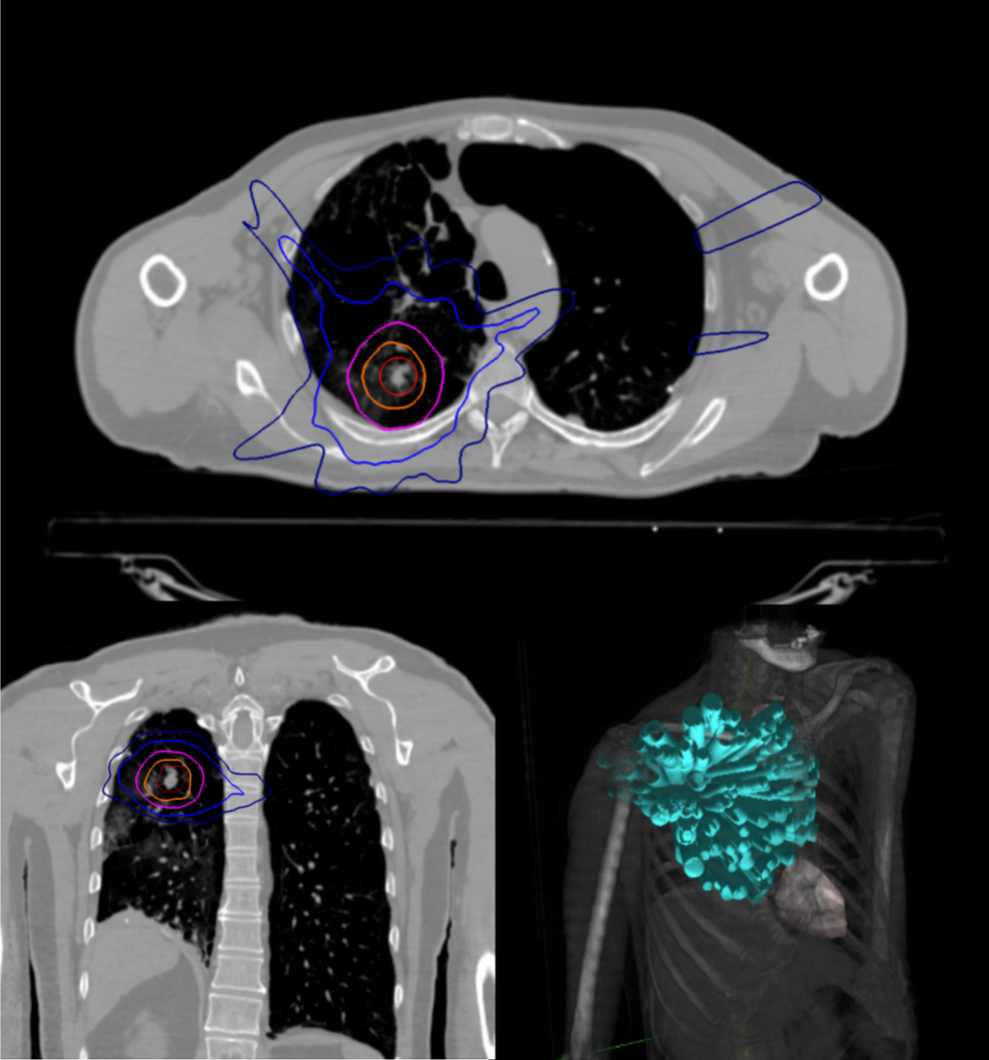
SBRT example plan for stage I NSCLC

Image-guided robotic SBRT with fiducial-based tracking is an innovative method of treating stage I NSCLC that allows for treatment beam movement in concert with patient respiratory motion, yielding extremely accurate dose delivery (Fig. 2 illustrates a fiducial-based SBRT plan). This allows for treatment margin reduction and minimization of radiation exposure to normal tissues including the lung, heart, and esophagus [14, 15]. This margin reduction comes without cumbersome patient immobilization devices used with other linac-based SBRT systems, which can be difficult to tolerate for patients with underlying pulmonary dysfunction and other medical comorbidities [15]. Our results are similar to those reported in the RTOG 0236 with 3-year primary local control rates and locoregional control rates of 91.1 versus 97.6 % and 84.1 versus 87.2 %, respectively [10]. Interestingly, the 5-year results of RTOG 0236 demonstrate continued excellent primary tumor control of 93 % but appreciably lower locoregional control of 62 % [16]. This is in contrast with our 5-year results showing a lower primary tumor local control of 87.6 % but higher locoregional control of 71.8 %.

Intriguingly, the results of the present study demonstrate a very low lobar failure rate with only one involved lobe recurrence observed during the follow-up period. The first explanation for these low in-lobe failure rates may be the result of differences in dose distribution, including possible variations in isodose line prescription. Indeed, inclusion of CT identified spiculations within the treatment field is variable, dependent on the oncologist/institution, and could result in differences in involved-lobe failure rates [17]. As a result, differences in dose fall off in this region may adequately sterilize microscopic spread within these spiculations and are incumbent upon the isodose line chosen. Second, utilization of intrafractional tumor tracking could allow for more accurate dose delivery to the tumor during treatment. The third explanation may be our careful exclusion of patients with additional concerning pulmonary nodules, which may incidentally be located in the involved lobe and eventually declare themselves as malignant. The fourth and perhaps most interesting reason for our low involved-lobe failure rate resides in the simplification of radiological follow-up due to the presence of fiducials. Post-SBRT pulmonary fibrosis can be quite substantial and often obscures the location of the initially treated primary lesion [18]. Fiducial marking of the treated tumor allows for accurate identification of the treated field on follow-up imaging, which is not feasible in other non-fiducial-based SBRT methods. As a result, the radiologist may more easily differentiate local recurrences from involved lobe failures.

Fiducial placement, however, does not come without risk. As we report, there were notably more symptomatic pneumothoraces observed early on in this study when the majority of patients underwent fiducial placement with CT guidance. Eleven of 29 (38 %) patients who underwent CT-guided fiducial placement developed symptomatic pneumothoraces requiring chest tube placement. Kothary et al. reported pneumothoraces as the most commonly observed postprocedural complication following placement of fiducials under percutaneous CT guidance, which occurred in 45 % of their cohort [19]. An alternative strategy was developed for placement of fiducials under bronchoscopic guidance with endobronchial ultrasound. Harley et al. reported this new technique with 43 consecutive patients who underwent fiducial placement using the endobronchial method [20]. Side effects reported were minimal with only one observed pneumothorax. Concordantly, pneumothorax risk at our institution dropped to zero following the transition to bronchoscopic placement. This technique not only has minimized morbidity but also affords an all-inclusive diagnostic approach in which the mediastinum can be staged, the primary tumor biopsied, and fiducials placed in a single procedure.

As we report here, a small but consistent decline in PFTs is noted in patients 1-year post-SBRT. Indeed, our understanding of pulmonary function changes following SBRT continues to evolve. Guckenberger et al. reviewed pretreatment and posttreatment PFTs in nearly 500 patients who were treated with SBRT for early-stage NSCLC [21]. They report statistically significant and progressive short-term (<6 months) and long-term (6 to 24 months) pulmonary function declines of 3.6 and 6.8 %, respectively, averaged over all patients and pulmonary function parameters. Analogously, we note a 5.7 % PFT decline averaged over all PFT values for the entire cohort at a median follow-up of approximately 1 year. An analysis of RTOG 0236 also found similar declines in FEV1 and DL_CO_ at 2 years of 5.8 and 6.3 %, respectively [22]. Conversely, Bishawi et al. reported no significant changes in FEV1 or FVC following SBRT treatment [23]. More recently, the University of Torino reported prospective data of 30 patients showing significant reductions in FEV1 and DL_CO_ following SBRT treatment at both 135 and 315 days of follow-up (3.21 and 6.32 %; 7.57 and 14.85 %, respectively) [24]. The consensus from the literature as well as our institutional data demonstrates that SBRT treatment is associated with progressive—albeit small—PFT changes, which are of unclear clinical impact and may also be explained by the natural history of patients’ underlying cardiopulmonary disease.

The close proximity of peripheral NSCLC tumors to the chest wall results in meaningful late toxicity following SBRT. In our cohort, we observed radiological evidence of rib fractures in those at-risk ribs in approximately one in five patients. Dunlap et al. reported toxicity data from 60 patients treated with SBRT to peripheral (defined as within 2.5 cm of the chest wall) primary or metastatic thoracic lesions [25]. Approximately 28 % of patients developed late grade 3 or higher chest wall pain, but only five of these patients had documented radiological rib fractures. Similarly, retrospective data from Voroney et al. found that 11 of 42 patients developed chest wall pain following treatment of peripheral early-stage NSCLC with three-fraction SBRT (total dose of 54 to 60 Gy) [26]. A total of nine patients were found to have rib fractures though two of these were asymptomatic. A Swedish retrospective review of 33 patients treated with SBRT to 45 Gy in 3 fractions identified 81 at-risk ribs defined as those receiving at least 21 Gy [27]. Of these at-risk ribs, 13 fractures (16 %) were found in seven patients. Undoubtedly, the chest wall should be considered a potential site for late SBRT-related toxicity, and this toxicity seems to manifest itself both as radiological rib fractures as well as non-specific chest wall pain. Despite the absence of ITV use in our study, rib fractures were not prominently reduced compared with historical data, though, given most respiratory motion is in the superior-inferior axis, this is not surprising. Ultimately, the optimal dose constraints for both rib and chest wall remain uncertain, and the appropriateness of these constraints are worrisome in the face of a potential detriment in tumor coverage and excess lung toxicity.

Our single institution retrospective review of patients with medically inoperable stage I NSCLC treated with SBRT demonstrates that comparable local control, locoregional control, and overall survival can be achieved with this novel fiducial-based technique. Limitations of the present study include its retrospective nature, the heterogeneity of SBRT fractionation schedules, and the small patient cohort. Additionally, due to its retrospective review, we were unable to correlate radiological rib fractures with clinical manifestations of chest wall syndrome. Several large-scale propensity-matched analyses have shown either equivalent or superior clinical outcomes with SBRT versus surgery [28, 29]. Although poor accrual has prevented definitive conclusions to be made from previous phase 3 studies of surgery versus SBRT in the operable population, a recent combined analysis of the STARS and ROSEL trials demonstrated a 3-year overall survival of 95 versus 79 % (*p* = 0.037) in SBRT and surgery groups, respectively [30]. As evidence of SBRT treatment efficacy, limited toxicity, and patient preference continues to mount in the medically inoperable population, future trials including VALOR and SABRTooth will directly compare the merits of SBRT versus surgery in the operable population [8, 31].

## Conclusion

Mediastinal evaluation, lesion biopsy, and fiducial placement by an interventional pulmonologist represent an all-inclusive and minimally morbid management strategy for the medically inoperable stage I NSCLC patient in preparation for SBRT treatment. Bronchoscopic fiducial placement clearly results in a lower risk of pneumothoraces requiring chest tube placement relative to CT-guided placement. There are observable PFT declines following SBRT treatment, but in this cohort, the only statistically significant decline was observed in DL_CO_. The long-term follow-up reported here demonstrates that despite reduced treatment margins and lack of patient immobilization, this tumor tracking method leads to clinically comparable outcomes with other linac-based SBRT approaches. Nevertheless, the reduction in irradiation volume did not translate into clinically obvious improvements in toxicity. Future prospective trials should explore the optimal SBRT technique for treating stage I NSCLC.

## Abbreviations

BED: Biological effective dose
DL_CO_: Diffusing capacity of lung for carbon monoxide
FEV: Forced expiratory volume
FVC: Forced vital capacity
GTV: Gross tumor volume
IDL: Isodose line
NSCLC: Non-small cell lung cancer
PTV: Planning target volume
SBRT: Stereotactic body radiation therapy
TLC: Total lung capacity
